# Prognostic value of serum γ-glutamyl transferase in unresectable hepatocellular carcinoma patients treated with transcatheter arterial chemoembolization combined with conformal radiotherapy

**DOI:** 10.3892/ol.2014.2456

**Published:** 2014-08-19

**Authors:** DONG CHEN, RENBEN WANG, XIANGJIAO MENG, HONGJIANG YAN, SHUMEI JIANG, RUI FENG, KUNLI ZHU, XIAOQING XU, XUE DOU, LINZHI JIN

**Affiliations:** 1 Department of Radiation Oncology, Shandong Cancer Hospital, Jinan, Shandong 250117, P.R. China; 2 School of Medicine and Life Sciences, University of Jinan-Shandong Academy of Medical Sciences, Shandong Cancer Hospital, Jinan, Shandong 250117, P.R. China

**Keywords:** prognostic value, serum γ-glutamyl transferase, hepatocellular carcinoma, transcatheter arterial chemoembolization, embolization, three-dimensional conformal radiotherapy

## Abstract

The detection of γ-glutamyl transferase (GGT) has previously been reported to be useful in the diagnosis in hepatocellular carcinoma (HCC). The aim of the present study was to investigate the baseline serum GGT levels in patients with intermediate HCC (Barcelona Clinic Liver Cancer stage B) following treatment with transcatheter arterial chemoembolization (TACE) combined with three-dimensional conformal radiotherapy (3DCRT). A total of 154 intermediate HCC patients with Child-Pugh grade A were retrospectively investigated. Receiver operating characteristic (ROC) analysis was used to determine the optimal threshold for the GGT serum levels, and univariate and multivariate analyses were used to establish the prognostic factors. The median overall survival (OS) time was 24.3 months. The optimal threshold for GGT was 85 U/L (sensitivity, 75.13%; specificity, 69.81%; and area under the ROC curve, 0.763). The one-, three- and five-year OS rates were 79.9, 49.7 and 17.2%, respectively, for patients with low GGT levels (≤85 U/l) and 52.3, 22.1 and 8.5%, respectively, for patients with high GGT levels (>85 U/l) (P=0.007). The results indicated that the serum GGT level was an independent prognostic factor (hazard ratio=2.32; P=0.007) for OS. Furthermore, in subgroups stratified according to serum α-fetoprotein, gross tumor volume and radiation dose, serum GGT was also found to correlate with OS (P<0.05). Therefore, the baseline GGT level may be a significant prognostic factor for intermediate HCC patients with Child-Pugh grade A following TACE combined with 3DCRT.

## Introduction

Worldwide, hepatocellular carcinoma (HCC) is the fifth most prevalent type of cancer and, after lung and stomach cancer, is the third most common cause of cancer-related mortalities (1). Resection and liver transplantation are generally regarded as curative treatments for early-stage HCC and have exhibited effective results (2,3). However, the majority of patients diagnosed with intermediate- to advanced-stage HCC receive only palliative treatment, such as transcatheter arterial chemoembolization (TACE). As the technology has developed, three-dimensional conformal radiotherapy (3DCRT) has allowed for high-dose radiation to be delivered to the target volume accurately, while minimizing the dose to normal liver tissues. TACE, combined with conventional external radiotherapy, has become the main treatment option for intermediate- to advanced-stage HCC, and the associated studies have reported safe and effective outcomes (4,5).

γ-glutamyl transferase (GGT) is a cell surface heterodimeric glycoprotein, which is routinely tested for in clinical examinations. It is a simple biological marker which can be easily obtained from the patient at a low cost. High expression is observed in the biliary epithelium, brain capillaries and kidney tubules (6). According to Griffith *et al* (7), serum GGT can be used as a diagnostic biomarker for hepatobiliary disease, and GGT has been confirmed to be a major prognostic factor for survival in cirrhosis (8). Studies have demonstrated that serum GGT can predict tumor response and survival after TACE and surgery (9,10); however, little is known regarding the prognostic role of GGT in treatment with combined TACE and 3DCRT. In the current study, 154 intermediate [Barcelona Clinic Liver Cancer (BCLC) stage B] (11) HCC patients were retrospectively investigated and the predictive value of the baseline serum GGT level with regard to overall survival (OS) was analyzed following the combined treatment.

## Patients and methods

### Study design

The current retrospective study was conducted at the Department of Radiation Oncology at Shandong Cancer Hospital (Jinan, China). The criteria for entry into this study were as follows: i) HCC confirmed by liver biopsy or with the clinical features defined by the American Association for the Study of Liver Diseases [persistently elevated α-fetoprotein (AFP) levels (>400 ng/ml) in conjunction with characteristic abdominal computed tomography (CT) or magnetic resonance imaging (MRI) with arterial phase enhancement and venous phase washout] (12); ii) all patients of intermediate stage (BCLC stage B) with Child-Pugh grade A according to the BCLC staging system (11); iii) Eastern Cooperative Oncology Group performance status of 0–1 (13); and iv) available follow-up data. The study protocol was approved by Shandong Tumor Prevention and Control Institutional Ethics Committee, Shandong Cancer Hospital and all patients provided written informed consent.

The clinical features of all patients included age, gender, tumor size, gross tumor volume (GTV), hepatitis virus infection, radiotherapy dose and total number of TACE treatments. The blood samples were obtained the morning prior to the TACE. Indicators of liver damage, including alanine transferase (ALT), GGT, albumin (ALB) and AFP, were systematically analyzed. The baseline imaging results (CT or MRI) of the liver were assessed within a week prior to TACE. For continuous variables, including age, GGT, tumor size, GTV and total number of TACE treatments, patients were divided into two groups according to the median values.

### TACE procedures

TACE was performed using the conventional Seldinger technique (14). Hepatic and superior mesenteric artery angiographies were performed to identify the tumor vessel anatomy, tumor staining and the tumor-feeding artery. The catheter was superselectively inserted into the tumor-feeding artery in as close proximity as possible to the tumor. Chemotherapeutic agents, including 1.0 g 5-fluorouracil and 80 mg cisplatin were infused, following which, an emulsion of 10 mg mitomycin C and 5–30 ml lipiodol was administered. The dosage of chemotherapeutic agents or lipiodol was selected based on the tumor size, liver function and routine blood analysis. For large tumors that were hypervasculature in nature, a gelatin sponge was used for the further embolization of the tumor-feeding artery. TACE was performed every 1.5–2.0 months if required, on the basis of the tumor response and patient health.

### 3DCRT procedure

3DCRT was performed two to four weeks after the final TACE course. A CT scan was initially performed for treatment planning. The patient position was fixed using vacuum casts in a supine position, with the arms raised above the head. GTV was delineated according to the primary lesion or lipiodol deposit from TACE. The clinical target volume was expanded by 5 mm on the basis of GTV and the planning target volume (PTV) was defined as GTV plus a 5-mm radial expansion, as well as a 10-mm craniocaudal expansion to account for daily setup error and respiratory organ motion (15). Organs at risk were also delineated, including the whole liver, non-target liver (whole liver minus PTV), stomach, kidney and spinal cord. Aided by the beam’s eye view, four to six coplanar or non-coplanar fields were designed. A cumulative dose-volume histogram was used to evaluate each treatment plan, and the target delineation was conducted by the same experienced oncologist. The median radiation dose was 45 Gy (range, 10–60 Gy) and the mean dose to normal liver was limited to ≤30 Gy.

### Evaluation of GGT and follow-up

The serum concentrations of GGT were analyzed using a Hitachi 917 machine (Roche Diagnostics, Mannheim, Germany). Tumor responses were evaluated with contrast-enhanced CT or MRI one month following TACE or 3DCRT. For patients without a complete response (CR), TACE was repeated. If the patients achieved a CR, contrast-enhanced ultrasound, AFP test, CT and MRI were performed within three months following the treatment, and then routinely performed every six months until December 2013. In addition, routine blood analysis was conducted and liver function and serum tumor markers were also analyzed.

### Statistical analysis

The software used for statistical analysis was SPSS 13.0 for Windows (SPSS Inc., Chicago, IL, USA). All consecutive results were presented as the mean ± standard deviation. Comparison of variables was performed by the Mann-Whitney U test, χ^2^ test or Fisher’s exact test. Variables that achieved statistical significance in the univariate analysis were subsequently included in a multivariate analysis using a stepwise forward Cox regression procedure to identify factors independently associated with mortality. OS was calculated as the interval between the time of the initiation of treatment and the time of mortality. The optimal threshold for GGT was identified by the receiver operating characteristic (ROC) curve, derived from a univariate logistic regression model predicting patient mortality prior to the median OS. This threshold served in all further uni- and multivariate analyses. Cumulative survival curves for each variable were obtained by using the Kaplan-Meier method and the difference was compared using the log-rank test. P<0.05 was considered to indicate a statistically significant difference.

## Results

### Prognostic factors affecting survival

**Figure 1:**
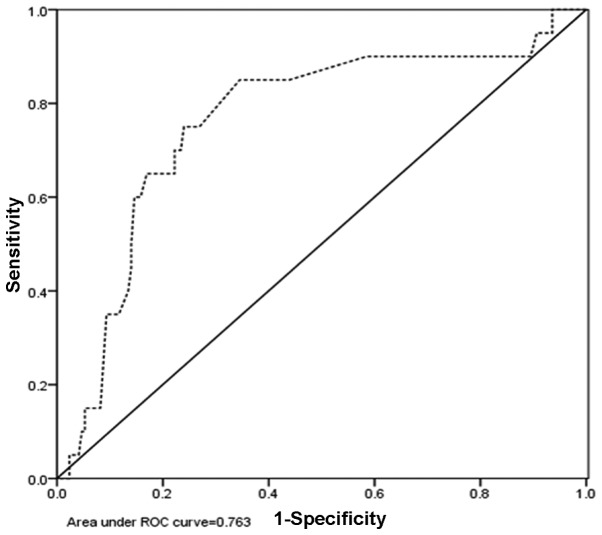
ROC curve derived from a univariate logistic regression model predicting patient mortality prior to the median overall survival time. The optimal threshold for γ-glutamyl transferase was 85 U/l. This resulted in a sensitivity of 75.13% and a specificity of 69.81%. Area under the ROC curve, 0.763; 95% confidence interval, 0.645–0.880. ROC, receiver operating characteristic.

A total of 154 patients with intermediate HCC (71 females and 83 males) between January 2004 and December 2010 were included in the study. The median age and GTV were 55 years (range, 23–71 years) and 200 cm^3^. At the time of the analysis, the median number of TACE procedures performed for all patients was four (range, one to 10). According to the ROC analysis (Fig. 1), the optimal threshold for GGT was 85 U/l. This resulted in a sensitivity of 75.13% and a specificity of 69.81% [area under the ROC curve, 0.763; 95% confidence interval (CI), 0.645–0.880]. Furthermore, 115 patients (74.7%) were included in the high GGT group, according to the cut-off level, and 39 patients (25.3%) were included in the low GGT group.

The baseline characteristics of the 154 patients are summarized in Table I. The results indicated that GGT levels (P=0.003), ALT levels (P=0.012), ALB levels (P=0.038), GTV (P=0.002), AFP levels (P=0.01), total number of TACE procedures (P=0.039) and radiation dose (P=0.044) were all associated with OS. Factors exhibiting a significant difference by univariate analysis were adopted when multivariate Cox proportional-hazards analysis was performed. The results demonstrated that GGT levels [P=0.001; hazard ratio (HR), 2.32; 95% CI, 1.133–3.643], GTV (P=0.007; HR, 1.263; 95% CI, 1.361–7.401), AFP levels (P=0.006; HR, 1.84; 95% CI, 1.218–3.059) and radiation dose (P=0.035; HR, 1.75; 95% CI, 1.157–2.998) were independent risk factors for OS (Table II). A comparison of the clinical results between patients with low and elevated GGT expression is summarized in Table III. The results indicated that patients with elevated GGT usually had higher serum ALT, AFP and total bilirubin levels, as well as lower ALB and shorter prothrombin time.

### OS of patients with various GGT levels

**Figure 2:**
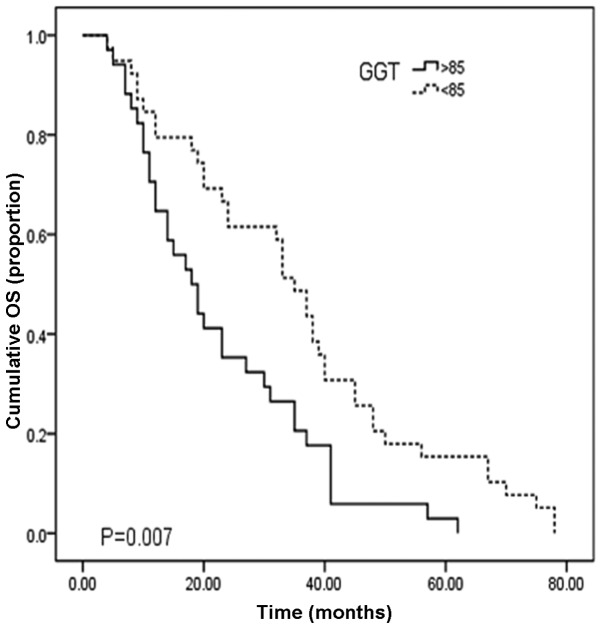
Cumulative OS curve of patients with low and high serum GGT levels (cut-off value was 85 U/l). The one-, three- and five-year OS rates were 79.9, 49.7 and 17.2 % for patients with low GGT levels (≤85 U/l), and 52.3, 22.1 and 8.5% for patients with high GGT levels (>85 U/L). OS, overall survival; GGT, γ-glutamyl transferase.

In Table IV, the median OS time following TACE combined with 3DCRT was 24.3 months (95% CI, 12.84–35.16), with one-, three- and five-year OS rates of 62.1, 27.5 and 10.9%, respectively. Fig. 2 shows the cumulative overall survival curve for patients with low (≤85 U/l) and high GGT levels (>85 U/l). For HCC patients with low GGT levels (n=39), the median OS time was 35.0 months (95% CI, 29.9–40.1) with 1-, 3- and 5-year survival rates of 79.9, 49.7 and 17.2%, respectively. For patients with high GGT levels (n=115), the median OS time was 18.0 months (95% CI 12.3–23.7) with 1-, 3-, and 5-year survival rates of 52.3, 22.1 and 8.5%, respectively. The OS time of low GGT patients was significantly longer than that of the elevated GGT group (Fig. 2; P=0.007).

**Figure 3:**
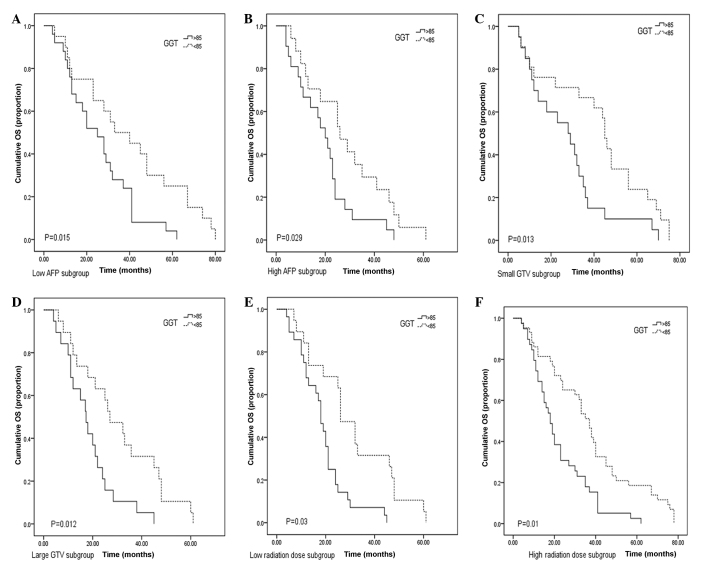
Kaplan-Meier estimates of OS rates for patients treated by transcatheter arterial chemoembolization with three-dimension conformal radiotherapy. The graphs represent the assessment of the prognostic role of GGT in subgroups, which were stratified according to (A and B) AFP levels (≤400 or >400 ng/ml, respectively), (C and D) GTV (≤200 or >200 cm^3^, respectively) and (E and F) radiation dose (≤45 or >45 Gy, respectively). OS, overall survival; AFP, α-fetoprotein; GTV, gross tumor volume; GGT, γ-glutamyl transferase.

Considering the effects of the high AFP levels, large GTV and high radiation dose on OS, these factors were stratified to further clarify the prognostic significance of GGT levels. The results demonstrated that serum GGT levels correlated with OS time in the subgroup of low (≤400 ng/ml) and high (>400 ng/ml) serum AFP levels (P=0.015 and 0.029, respectively; Fig 3A and B). When the results were stratified according to GTV, patients with low serum GGT levels had a longer OS time compared with that of the high GGT level group (P=0.013 and 0.012, respectively; Fig 3C and D). For the patients receiving a low radiation dose (≤45Gy), those with high GGT levels exhibited a shorter OS time compared with that of the low GGT group (P=0.03; Fig. 3E). In the high radiation dose group, a significant difference was also observed in OS time between patients with low and high GGT levels (P=0.01; Fig. 3F).

## Discussion

Measurement of GGT levels has been investigated and developed as a liver function test for several decades (14,15). Hann *et al* (18) reported that serum GGT levels may predict HCC risk and mortality in hepatitis B virus (HBV) patients. Guiu *et al* (19) suggested that a serum GGT level of ≥165 U/L was associated with shorter time to treatment failure and OS time following TACE. Zhang *et al* (10) revealed that the predictive value was stable, and even higher, when a threshold of between 60 and 300 U/L was used in a large retrospective study (277 patients). Furthermore, elevation of GGT levels was confirmed as a predictor of poor clinical outcome for intrahepatic cholangiocarcinoma patients (19). However, the correlation between GGT levels and TACE combined with 3DCRT remains unexplored. In the current study, the results demonstrated that GGT levels of >85 U/l were associated with a shorter OS time (P=0.007). The optimal threshold of GGT levels (85 U/l) was identified by the ROC analysis (Fig. 1), derived from a univariate logistic regression model predicting patient mortality prior to the median OS.

The molecular mechanisms of GGT in HCC development remain unclear. It has been suggested that functions of the oxidative stress pathways in cellular response may mediate the role of GGT in tumorigenesis (21). The membrane-bound enzyme, GGT, catalyzes the degradation of extracellular glutathione (GSH), making the component amino acids available for the resynthesis of intracellular GSH (6). GSH can protect cells from damage induced by oxidants generated during normal metabolism. There is extensive evidence to suggest that GGT and GSH can cooperatively generate free radicals, subsequently leading to lipid peroxidation (18,22,23). An additional explanation for the predictive nature of GGT on OS of HCC patients in the current study is the significant implication of lipid peroxidation and other metabolisms in the tumorigenesis of a number of malignancies, including HCC (24,25). Furthermore, an increased level of intracellular GSH often correlates with resistance to platinum-based drugs (26). Daubeuf *et al* (27) revealed that GGT activity may affect the cytotoxicity of platinum drugs in two ways: i) Following a reaction with the thiol group of cysteinylglycine, cisplatin can be detoxified extracellularly; or ii) in the case of carboplatin, GCT initiates the supply of GSH precursors, which subsequently increases the intracellular level of the tripeptide and provides enhanced defensive mechanisms to the cell. In the current study, cisplatin was the chemotherapeutic agent used during the TACE procedure. This may also explain the longer OS time of patients with low GGT levels (≤85 U/l) compared with those with high GGT levels (>85 U/l) as increased levels of intracellular GSH are often found to correlate with resistance to platinum-based drugs and a high level of GGT is associated with a higher concentration of GSH. Furthermore, in subgroups stratified according to serum AFP levels, GTV and radiation dose, GGT levels still had the power to discriminate patients with good results from those with poor outcomes.

In the present study, univariate analysis indicated that ALT levels, GTV, AFP levels, radiation dose and the number of TACE procedures all correlate with OS. In the multivariate analysis, only radiation dose, GTV and AFP levels were independent prognostic factors. The number of TACE procedures were not an independent predictive factor as different numbers of TACE were performed for each patient until the iodized oil deposited the whole tumor.

To date, radiotherapy technology has evolved markedly and is significant in the treatment of HCC. Kouloulias *et al* (28) reported that a high radiation dose (50–52 Gy) of 3DCRT can achieve a high local control rate in advanced HCC patients and inferior vena cava tumor thrombosis. In the current study, patients receiving a radiation dose of >45 Gy may achieve improved survival compared with those receiving a low radiation dose (P=0.035). According to Son *et al* (29) a large volume of liver receiving radiotherapy may lead to radiation-induced liver disease (RILD), which may result in hepatic failure and mortality. The authors suggested that in order to reduce the risk of RILD, the total liver volume receiving <18 Gy must be >800 cm^3^; therefore, sparing more normal liver during radiotherapy is essential for HCC patients. In the current study, longer survival was observed in patients with smaller GTV (≤200 cm^3^) compared with that of larger GTV (P=0.013).

Cell proliferation and angiogenesis are promoted by AFP, as well as the increased resistance of cells toward tumor necrosis factor-associated, apoptosis-inducing ligand-induced apoptosis (30–32). It is well reported that AFP levels are a significant prognostic factor for patients following radiofrequency ablation and resection (33,34). Tsai *et al* (35) and Kohles *et al* (36) demonstrated that AFP levels can be used as a biomarker to predict poor response following TACE. The current study indicated that serum AFP levels were an independent prognostic factor (P=0.006) for intermediate HCC patients treated with TACE combined with 3DCRT.

The present study had certain limitations, including the retrospective design and small number of patients. Therefore, further studies investigating larger patient populations are required to validate the results of the study.

In conclusion, the results presented in this study demonstrated that the baseline GGT levels of intermediate HCC patients with Child-Pugh grade A is an independent prognostic factor for OS following TACE combined with 3DCRT. In additon, the results of the present study may aid to predict outcomes for patients and may also be used to guide individualized treatment for HCC patients that receive TACE in combination with 3DCRT.

## Figures and Tables

**Table I:** Univariate analysis of factors associated with overall survival.

		Overall survival rate, %			
			
Risk factors	n	1-year	3-year	5-year	P-value
Age, years					0.772
≤55	60	70.0	31.7	13.3	
>55	94	58.5	21.3	9.6	
Gender					0.144
Male	83	71.1	42.2	14.5	
Female	71	46.5	12.7	5.6	
Total bilirubin, μmol/l					0.586
≤17.1	60	61.7	31.7	8.3	
>17.1	94	43.6	19.1	7.4	
GGT, U/l					0.003
≤85	39	79.5	48.7	17.9	
>85	115	52.2	21.7	8.7	
Prothrombin time, sec					0.388
≤14	121	66.9	38.8	9.1	
>14	33	33.3	15.2	3.0	
ALT, U/l					0.012
≤40	74	63.5	31.1	9.5	
>40	80	45.0	20.0	8.8	
ALB, g/l					0.038
≤35	43	32.6	18.6	4.7	
>35	111	57.7	33.3	7.2	
AFP, ng/ml					0.010
A≤400	92	60.9	51.1	14.1	
B>400	62	40.3	11.3	3.2	
GTV, cm^3^					0.002
≤200	73	72.6	60.3	24.7	
>200	81	61.7	27.2	14.8	
Radiation dose, Gy					0.044
≤45	82	56.1	18.3	4.9	
>45	72	61.1	36.1	16.7	
TACE, n					0.039
1–4	70	61.4	11.4	8.6	
>4	84	64.3	30.9	12.9	
HBV					0.054
Positive	89	49.4	20.2	4.5	
Negative	65	50.8	38.5	12.3	

GGT, γ-glutamyl transferase; ALT, alanine transferase; ALB, albumin; AFP, α-fetoprotein; GTV, gross tumor volume; TACE, transcatheter arterial chemoembolization; HBV, hepatitis B virus.

**Table II:** Multivariate analysis of factors associated with overall survival.

Risk factors	Hazard ratio	95% CI	P-value
GGT (≤85 vs >85 U/l)	2.320	1.133–3.643	0.001
ALT (≤40 vs >40 U/l)	1.263	0.599–2.092	0.545
ALB (≤35 vs >35 g/l)	0.721	0.509–1.021	0.065
GTV (≤200 vs >200 cm^3^)	1.263	1.361–7.401	0.007
TACE, n	0.648	0.381–1.101	0.109
Radiation dose, Gy	1.750	1.157–2.998	0.035
AFP (≤400 vs >400 ng/ml)	1.840	1.218–3.059	0.006

GGT, γ-glutamyl transferase; ALT, alanine transferase; ALB, albumin; GTV, gross tumor volume; TACE, transcatheter arterial chemoembolization; AFP, α-fetoprotein; CI, confidence interval.

**Table III:** Comparison of clinicopathological factors between patients with low and high γ-glutamyl transferase levels.

Risk factors	Low GGT(≤85 U/l)	High GGT (>85 U/l)	P-value
Gender, n			0.173
Male	23	60	
Female	27	44	
Age, years	50.2±12.1	51.7±14.5	0.087
ALT, U/l	39.7±18.9	57.3±38.5	0.023
HBV			0.088
Positive	24	65	
Negative	26	39	
AFP, ng/ml	3112.1±3840.3	21831.2±18723.5	0.020
Prothrombin time, sec	12.53±1.75	11.22±2.24	0.238
ALB, g/l	40.1±5.8	35.3±4.1	0.185
Total bilirubin, μmol/l	16.8±6.8	18.7±8.7	0.021

Data are presented as the mean ± standard deviation. ALT, alanine transferase; HBV, hepatitis B virus; AFP, α-fetoprotein; ALB, albumin; GGT, γ-glutamyl transferase.

**Table IV:** Different γ-glutamyl transferase levels associated with OS.

GGT	Median OS, months	OS rate, %			95% CI

		1-year	3-year	5-year	
All patients	24.3	62.1	27.5	10.9	12.8–35.2
≤85 U/l	35.0	79.9	49.7	17.2	29.9–40.1
>85 U/l	18.0	52.3	22.1	8.5	12.3–23.7

CI, confidence interval; OS, overall survival.
